# Enhancement of PD-L1-attenuated CAR-T cell function through breast cancer-associated fibroblasts-derived IL-6 signaling via STAT3/AKT pathways

**DOI:** 10.1186/s13058-023-01684-7

**Published:** 2023-07-21

**Authors:** Nisa Chuangchot, Pranisa Jamjuntra, Supaporn Yangngam, Piriya Luangwattananun, Suyanee Thongchot, Mutita Junking, Peti Thuwajit, Pa-Thai Yenchitsomanus, Chanitra Thuwajit

**Affiliations:** 1 Department of Immunology, Faculty of Medicine Siriraj Hospital, Mahidol University, Bangkok, 10700 Thailand; 2 Siriraj Center of Research Excellence for Cancer Immunotherapy, Research Department, Faculty of Medicine Siriraj Hospital, Mahidol University, Bangkok, 10700 Thailand; 3 Division of Molecular Medicine, Research Department, Faculty of Medicine Siriraj Hospital, Mahidol University, Bangkok, 10700 Thailand

**Keywords:** Cancer-associated fibroblast, IL-6, PD-L1, Folate receptor-*α*, CAR-T cell, Doxorubicin

## Abstract

**Background:**

Carcinoma-associated fibroblasts (CAFs) play a critical role in cancer progression and immune cell modulation. In this study, it was aimed to evaluate the roles of CAFs-derived IL-6 in doxorubicin (Dox) resistance and PD-L1-mediated chimeric antigenic receptor (CAR)-T cell resistance in breast cancer (BCA).

**Methods:**

CAF conditioned-media (CM) were collected, and the IL-6 level was measured by ELISA. CAF-CM were treated in MDA-MB-231 and HCC70 TNBC cell lines and si*IL-6* receptor (IL-6R) knocked down (KD) cells to determine the effect of CAF-derived IL-6 on Dox resistance by flow cytometry and on increased PD-L1 through STAT3, AKT and ERK1/2 pathways by Western blot analysis. After pre-treating with CM, the folate receptor alpha (FR*α*)-CAR T cell cytotoxicity was evaluated in 2D and 3D spheroid culture assays.

**Results:**

The results showed a significant level of IL-6 in CAF-CM compared to that of normal fibroblasts (NFs). The CM with high IL-6 level significantly induced Dox resistance; and PD-L1 expression through STAT3 and AKT pathways in MDA-MB-231 and HCC70 cells. These induction effects were attenuated in si*IL-6R* KD cells. Moreover, the TNBC cell lines that were CM-treated with STAT3 and an AKT inhibitor had a reduced effect of IL-6 on PD-L1 expression. BCA cells with high IL-6 containing-CM treatment had resistance to cancer cell killing by FR*α* CAR-T cells compared to untreated cells.

**Conclusion:**

These results highlight CAF-derived IL-6 in the resistance of chemotherapy and T cell therapy. Using inhibitors of IL6-STAT3/AKT-PD-L1 axis may provide a potential benefit of Dox and CAR-T cell therapies in BCA patients.

**Supplementary Information:**

The online version contains supplementary material available at 10.1186/s13058-023-01684-7.

## Introduction

Breast cancer (BCA) is the most common cancer of women worldwide [1]. The 2020 global cancer statistics estimate 2.3 million new cases in women [2]. BCA is clustered into four subtypes, including Luminal A, Luminal B, HER2-positive, and triple-negative BCA (TNBC) [3]. Although increased early screening and understanding of molecular mechanisms of metastasis leads to more effective prognosis and treatment, some patients have relapses and chemoresistance, especially in the TNBC [4].

The tumor microenvironment (TME) consists of tumor cells, cancer-associated fibroblasts (CAFs), endothelial cells, extracellular matrix, and immune cells [5]. CAFs, the most abundant cells in TME, secrete substances such as transforming growth factor-*β* (TGF-*β*), vascular endothelial growth factor (VEGF), and interleukin-6 (IL-6) which can promote cancer progression [6–9]. CAF-derived substances regulate epithelial-mesenchymal transition and promote drug resistance by secreting IL*-*6 [10, 11]. In addition, fibroblast activation protein (FAP) *α*1-positive CAFs drive immunosuppression [12–14]. These facts declare the importance to investigate CAF-mediated immunosuppression and the involved mechanisms.

IL-6 is released by various cells including cancer cells, CAFs, and immune cells [15]. IL-6/IL-6 receptor (IL-6R) interaction activates Janus kinase (JAK) tyrosine kinases leading to phosphorylating the signal transducer and activator of transcription 3 (STAT3) [15]. This interaction can phosphorylate mitogen-activated protein kinases (MAPK)/ERK leading to enhance cell growth [16]. The phosphoinositol-3 kinase (PI3K)/AKT pathway is induced by IL-6 following JAK phosphorylation. The PI3K/AKT phosphorylation induces gene expression by activating NF-κB to promote anti-apoptosis [17, 18].

Programmed death ligand 1 (PD-L1), the ligand for programmed death 1 (PD-1), is overexpressed in cancer cells after being induced by cytokines [19]. PD-L1 expression in cancer cells leads to immune escape, drug resistance, and cancer metastasis [20]. The PD-L1/PD-1 interaction attenuates T cell function via T cell apoptosis [21]. Patient-derived hepatocellular carcinoma (HCC)-CAFs secrete IL-6 which induces PD-L1 expression on neutrophils via the STAT3 pathway [22]. The PD-1/PD-L1 immune checkpoint inhibitor was approved by the US Food and Drug Administration (FDA) to treat advanced TNBC tumor-expressed PD-L1 [23]. Immunoregulatory roles of CAF-derived IL-6 in promoting drug resistance and inducing T cell dysfunction through IL-6-mediated PD-L1 expression have not been well-addressed in BCA.

In this study, the IL-6 ELISA assay of BCA-derived CAFs was performed to identify IL-6 levels as a major component in CAFs. The roles of IL-6 signaling to exert doxorubicin (Dox) resistance and response to folate receptor (FR)*α*-chimeric antigen receptor (CAR)-T cell killing actions were evaluated in parental and IL-6R -transient knocked down TNBC cell lines, with or without specific inhibitors against signaling molecules. The findings highlight the role of CAFs-derived IL-6 in mediating Dox resistance in MDA-MB-231 and HCC70 TNBC cell lines. It can also reduce FR*α*-CAR T cell function via the upregulation of PD-L1 through the STAT3/AKT pathway. Interestingly, using either blocking IL-6/IL-6R signaling or PD-1/PDL1 inhibitors may serve as a therapeutic target for sensitizing cancer cells to CAR-T cell treatment and restoring BCA cells to Dox treatment.

## Materials and methods

### Cell culture

MDA-MB-231 and HCC70 cells from ATCC (Manassas, VA) were genetically engineered to express mWasabi-luciferase protein [24]. The Lenti-X™-293T cells were from Takara Bio (Takara Bio, San Jose, CA). Lenti-X™-293T and MDA-MB-231 were maintained in DMEM (Gibco; Thermo Fisher Scientific, MA), while HCC70 was cultured in RPMI1640 (Gibco). CAFs were isolated from BCA patients following the protocol approved by Siriraj Institutional Review Board (COA no. Si 329/2017). CAFs and normal fibroblasts (NFs) [normal breast fibroblast (BNF) and human dermal fibroblast (HDF)] were cultured in DMEM/F12 (Gibco). All complete media was supplemented with 10% fetal bovine serum (FBS; Gibco) and 1% penicillin–streptomycin (Sigma-Aldrich, MA) and incubated with cells at 37˚C with 5% CO_2._

### Immunocytochemistry staining for fibroblast markers

Cells were incubated with anti-panCK (1:200, sc-8018; Santa Cruz, CA), anti-VIM (1:500, sc-6260; Santa Cruz), anti-ASMA (1:200, A5228; Sigma, MA), and anti-FAP (1:100, ab53066; Abcam, Cambridge, UK) at RT for 3 h. The anti-mouse IgG-Cy3 (1:2,000, 115-166-071; Jackson ImmunoResearch Laboratories, PA) or anti-rabbit IgG-FITC (1:2,000, ab6717; Abcam) was applied at RT for 1 h. The anti-CD10-FITC (1:5, 21270103; Invitrogen, Thermo Fisher Scientific, MA) and anti-GPR77 (1:30, 342402; Biolegend, CA) were incubated with cells at 4°C overnight. The nuclei were stained with Hoechst dye (1:1,000; Invitrogen). The fluorescence signals were captured with a confocal microscope (LSM800, Carl Zeiss Microscopy, Jena, Germany).

### CAFs conditioned-media collection

Conditioned-media (CM) was collected from nine CAFs, namely PC-B-004, PC-B-044, PC-B-053, PC-B-099, PC-B-120, PC-B-130, PC-B-132, PC-B-140, PC-B-142 and 2 NFs cultured in 10% FBS containing DMEM/F12 until 85–90% confluency before replacing it with 1% FBS containing DMEM/F12. CM was then centrifuged at 2000 g at 4°C for 5 min. The supernatant was stored at -80˚C.

### IL-6 measurement by enzyme-linked immunosorbent assay

The CM-derived IL-6 was detected by ELISA according to the instructions (R&D, MN). Absorbance values at 450 nm were determined with a plate reader (Epoch-Microplate-BioTek, CA). IL-6 concentration was determined by the line-curve fitting of standard results.

### IL-6R knockdown by small interfering RNA

The 2 × 10^5^ cancer cells were cultured in a complete medium overnight. The transfection solution was prepared by mixing OptiMEM-I (Gibco) with either 160 pmol si*IL-6R* or Lipofectamine 3000 (Invitrogen), while a mock control was performed in the same manner without si*IL-6R*. The si*IL-6R* knockdown (KD) cells were stained with anti-IL-6R antibody (1:50, sc-373708; Santa Cruz, CA), followed by anti-mouse PE antibody (1:100, 22549814; ImmunoTools, Friesoythe, Germany) at 4°C for 1 h. The IL-6R level was checked by flow cytometry (Beckman Coulter Life Sciences, CA). Data were analyzed using FlowJo 10 software (Flowjo, LLC, OR).

### Drug cytotoxicity using annexin V-APC/ PI staining

Cancer cells were treated with CM with or without 5 μM Dox (IC_50_) (Sigma-Aldrich). After 48 h, cancer cells were stained by Annexin V-APC/propidium iodide (1:100, ImmunoTools) at 4°C for 1 h and then analyzed by flow cytometry (Beckman Coulter). The cell apoptosis was analyzed using FlowJo 10 software (Flowjo, LLC).

### Western blot analysis

Cells were treated with various CM or 50 ng/ml recombinant human IL-6 (rhIL-6, 206-IL-050, R&D) for 48 h to detect PD-L1 and for 30 min to detect signaling pathway proteins. After lysing with RIPA buffer (Invitrogen, Thermo Fisher Scientific, MA) and quantitating protein by Bradford kits (Bio-Rad, CA), 40 μg protein was loaded onto 10% SDS-PAGE and transferred to PVDF membrane (Bio-Rad). After blocking with 5% skim milk for 1 h, the membrane was incubated with the anti-human PD-L1 (1:500, ab205921; Abcam, Cambridge, UK) at 4°C overnight. The HRP-conjugated anti-rabbit (1:5000, ab6721; Abcam) or anti-mouse secondary antibodies (1:5000, ab6789; Abcam) were then incubated at RT for 1 h.

Cells were pre-treated with 2 μM AKT inhibitor (21597; Chaman Chemical, MI) or 10 μM Stattic (14590; Chaman Chemical) for 2 h before treating with CM for 48 h. The anti-STAT3 (1:1000, 9139; Cell Signaling Technology, MA), anti-pSTAT3 (1:2000, ab76315; Cell Signaling), rabbit anti-AKT (1:1000, 9272, Cell Signaling), rabbit anti-pSTAT3 (1:1000, 9275; Cell Signaling), anti-ERK1/2 (1:1000, 9102; Cell Signaling), and anti-phosphoERK1/2 (1:1000, 9101; Cell Signaling) followed by HRP-conjugated anti-rabbit (1:5000, ab6721; Abcam) or anti-mouse (1:5000, ab6789; Abcam) were used. The ECL (Invitrogen) was visualized under Gel Document (Syngene, Cambridge, UK). The protein intensity was measured by ImageJ software version 1.48v. The *β*-actin was used to normalize the total loading protein using anti-*β*-actin (1:20,000, sc-47778; Santa Cruz).

### Production and characterization of FR*α*-CAR T cells

The production of FR*α*-CAR-T cells from healthy donor blood was previously described [25]. The lentiviral particles were quantified via titration in SupT1 cells [26]. Blood donors provided written consent under SIRB ethical approval (COA no. Si 225/2022). T cells were transduced by FR*α*-CAR lentiviruses at a multiplicity of infection (MOI) of 50 using 100 μg/ml protamine sulfate (Sigma-Aldrich) and maintained in AIM-V (Gibco) with 5% human AB serum (Sigma-Aldrich), 20 ng/ml of IL-2 (ImmunoTools), 10 ng/ml of IL-7 (ImmunoTools) and 20 ng/ml of IL-15 (ImmunoTools) for 3 d.

T cells were phenotyped using eFluor™450-anti-human CD3 (1:100, 48–0038-42; Invitrogen), APC-conjugated anti-human CD4 (1:100, 21270046; ImmunoTools), and PerCP-anti-human CD8 (1:100, 21810085; ImmunoTools). The FR*α*-CAR expression was determined by Pierce™ biotinylated protein-L (1:100, 29997; Invitrogen) and AlexaFlour®488-conjugated streptavidin (1:500, S32354; Invitrogen), then analyzed using flow cytometry (Beckman Coulter). Data were analyzed using FlowJo 10 software (Flowjo, LLC).

### 2D and 3D culture cancer cell killing assays

For 2D culture, the mWasabi-luciferase-expressing target cancer cells (T) were treated with CM for 48 h. After removing the CM, the effector FR*α*-CAR effector T cells (E) were added at E:T ratios of 0.5:1, 1:1, and 2.5:1 for 24 h. Fluorescence images were taken under fluorescence microscopy (Olympus, Tokyo, Japan). The fluorescence intensity (FI) of target cells was detected (485/515 nm) using SynergyH1-Hybrid Reader (BioTek-Agilent, CA). The non-fluorescence-expressing cell lines were used as a blank control, while the target alone (control group) was used to normalize with all conditions regarded as no cytolysis (FI = 1). The formula of relative FI = (FI of test-blank)/ (FI of control-blank).

For 3D culture, the mWasabi-luciferase-expressing cells were seeded into an ultra-low attachment 96-well plate (CLS7007; Corning, MA) in 2.5% Matrigel® (354234, Corning). The spheroids were treated with CM on day 2 and cultured for 2 more days. Effector cells were labeled with CellTracker™ Orange CMRA (C34551; Invitrogen) before adding to target cells at E:T ratios of 2.5, 5:1, and 10:1, and then co-cultured for 4 d. Fluorescence images were detected by fluorescence microscopy (Olympus) and analyzed by CellSense Standard program version 1.15 (Olympus). The FI was analyzed by the ImageJ software 1.48v (http://rsbweb.nih.gov/ij/).

### Statistical analysis

Data were analyzed using one-way analysis of variance (ANOVA) or Student’s *t* test. All statistical calculations were performed with GraphPad Prism software version 5 (GraphPad Software Inc., CA). The data are shown as mean ± SD and considered statistically significant when *P* < 0.05.

## Results

### Expression of fibroblast markers

**Fig. 1:**
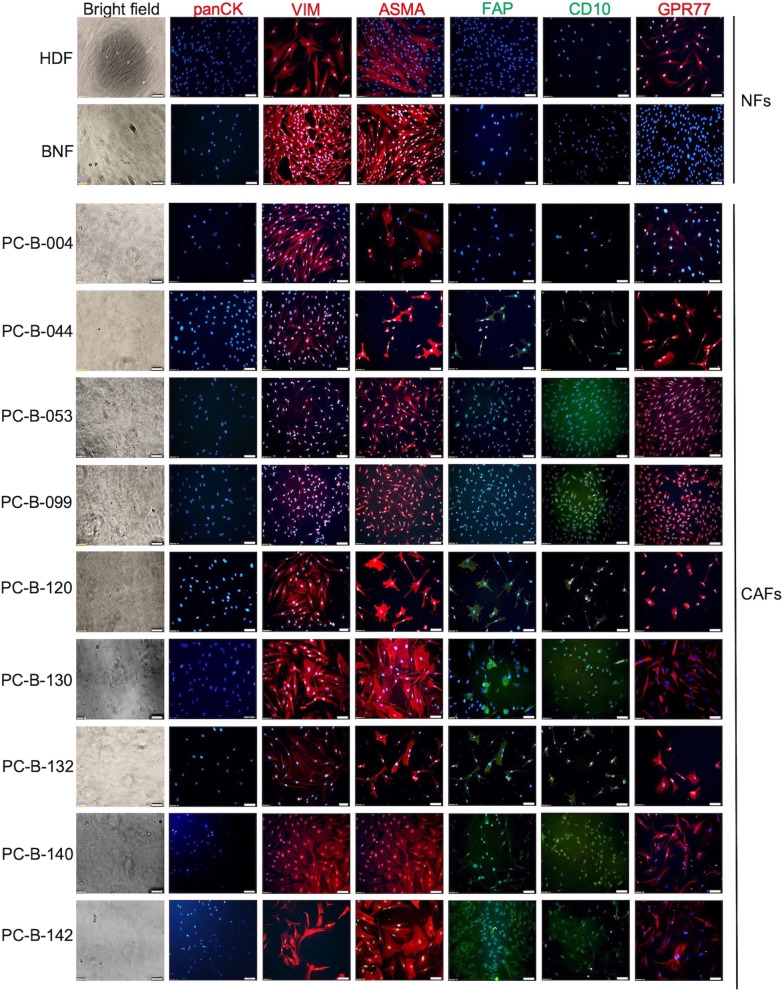
Characterization of primary culture fibroblasts. Immunofluorescence using antibodies directed to specific markers for epithelial cells (panCK, red fluorescence), fibroblast intermediate filament (VIM, red fluorescence), myofibroblasts (ASMA, red fluorescence), fibroblast-associated protein (FAP, green fluorescence), and chemotactic receptor (CD10, green fluorescence; GPR77, red fluorescence) of different primary culture fibroblasts and normal fibroblasts (HDF and BNF). The nuclei were counterstained with Hoechst. All images were captured at 200 × magnification, scale bars = 50 μm. NFs, normal fibroblasts; HDF, human dermal fibroblast; BNF, breast normal fibroblast; CAFs, cancer-associated fibroblasts; panCK, pan-cytokeratin; VIM, vimentin; ASMA, alpha-smooth muscle actin; FAP, fibroblast activation protein; CD10, a cluster of differentiation 10; GPR77, G protein-coupled receptor 77

CAFs showed the absence of CK epithelial markers, but the presence of VIM and ASMA mesenchymal markers [13]. FAP is used to classify aggressive CAF subtypes, while CD10/GPR77 markers are associated with drug resistance [27]. The results exhibited that all nine CAFs were negative for panCK and positive for both VIM and ASMA (Fig. 1). Among these nine CAFs, eight were FAP-positive CAFs (8/9 = 89%), PC-B-044, PC-B-053, PC-B-099, PC-B-120, PC-B-130, PC-B-132, PC-B-140, and PC-B-142, and one FAP-negative CAF (PC-B-004). All CAFs expressed GPR77 and eight CAFs except PC-B-004 expressed CD10. Notably, BNF showed negative FAP, CD10, and GPR77, while HDF was negative for FAP/CD10 and positive for GPR77.

### CAFs secreted high IL-6 levels and CAF-derived IL-6 induced PD-L1 expression in TNBC cells

**Fig. 2:**
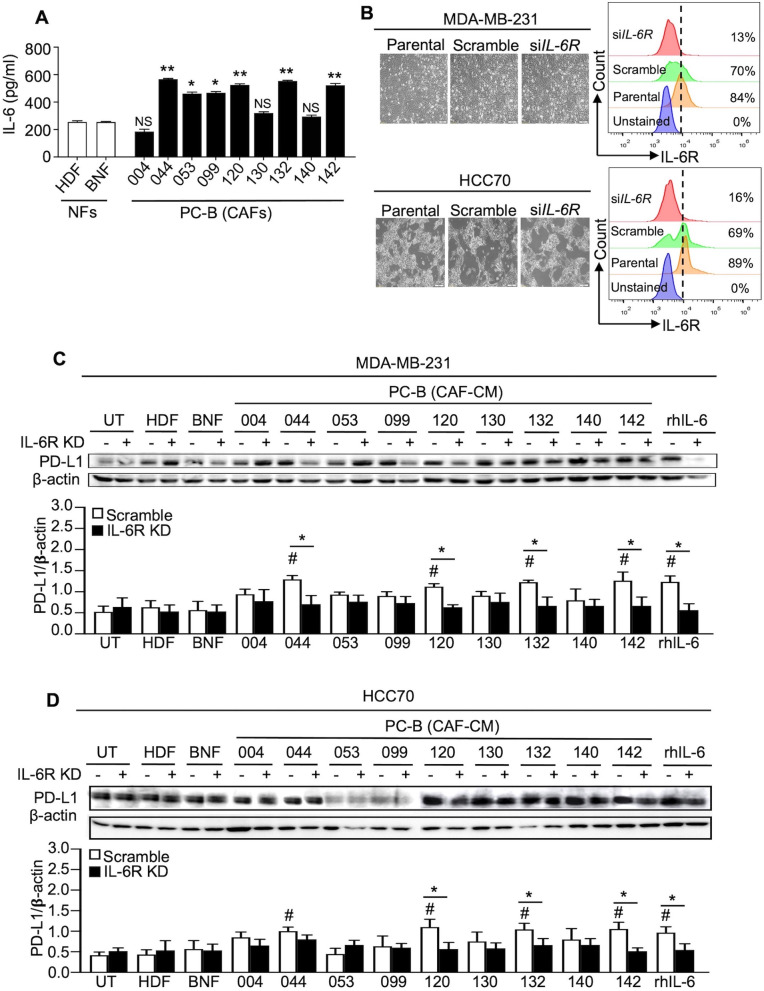
CAF-derived IL-6 and its effect on PD-L1 expression. **A** The level of IL-6 in CMs of different NFs and CAFs. Bar graphs represent the mean ± SD of three independent experiments. *P < 0.05, **P < 0.01 compared to BNF using one-way ANOVA. NS, no significance. **B** The representative morphology of parental MDA-MB-231 and HCC70 cells, scramble, and si*IL-6R* KD cells and the representative histogram of IL-6R detection by flow cytometry. **C**, **D** Effect of CAF-derived IL-6 on PD-L1 in MDA-MD-231 and HCC70 scramble and si*IL-6R* KD cells determined by Western blot analysis. *β*-actin was used as a housekeeping reference protein for semiquantitative analysis. Bar graphs represent the mean ± SD of three independent experiments. ^#^*P* < 0.05 compared to UT condition using one-way ANOVA. *P < 0.05 compared within group using Student's t test. CMs, conditioned-media; NFs, normal fibroblasts; HDF, human dermal fibroblasts; BNF, breast normal fibroblast; CAFs, cancer-associated fibroblasts; NS, no significance; IL-6R KD, IL-6R knocked down; UT, untreated cells; rhIL-6, recombinant human IL-6

Only IL-6 showed a significant increase in CAF-CM compared to CM from normal fibroblasts isolated from ovarian tissues (Additional file 1: Supplementary Method and Fig. S1). ELISA results confirmed that six of nine CAF-CM (67%) had significantly higher levels than BNF-CM (*P* < 0.05), which was defined as the high IL-6 CM (PC-B-044, PC-B-053, PC-B-099, PC-B-120, PC-B-132, and PC-B-142) (Fig. 2A). In contrast, three CMs (PC-B-004, PC-B-130, and PC-B-140) were defined as the low IL-6 CM.

The IL-6R in si*IL-6R* KD MDA-MB-231 was 13% and 16% in si*IL-6R* KD HCC70 cells compared to those of scramble cells (69% in MDA-MB-231, 62% in HCC70 cells) (Fig. 2B). The levels of IL-6R in the scramble and parental cells were not different. No morphological and viable cell changes of si*IL-6R* KD cells were observed in both cells compared to those of parental and scramble cells.

The role of CAF-derived IL-6 in inducing PD-L1 expression was observed in MDA-MB-231 (Fig. 2C) and HCC70 cells (Fig. 2D). NF-CM treated scramble cells showed no significant differences in PD-L1 compared to that of untreated cells in both cells. The rhIL-6-treated scramble cells significantly induced PD-L1 compared to that of BNF-CM (*P* < 0.05). Treating-scramble cells with high IL-6 CM (PC-B-044, PC-B-120, PC-B-132, and PC-B-142) significantly induced PD-L1 in both BCA cells compared to those of BNF-CM (*P* < 0.05). Interestingly, si*IL-6R* KD cells showed no response to high IL-6-CM-induced PD-L1 expression in both cell lines.

### The CAF-derived IL-6 induced Dox resistance

**Fig. 3:**
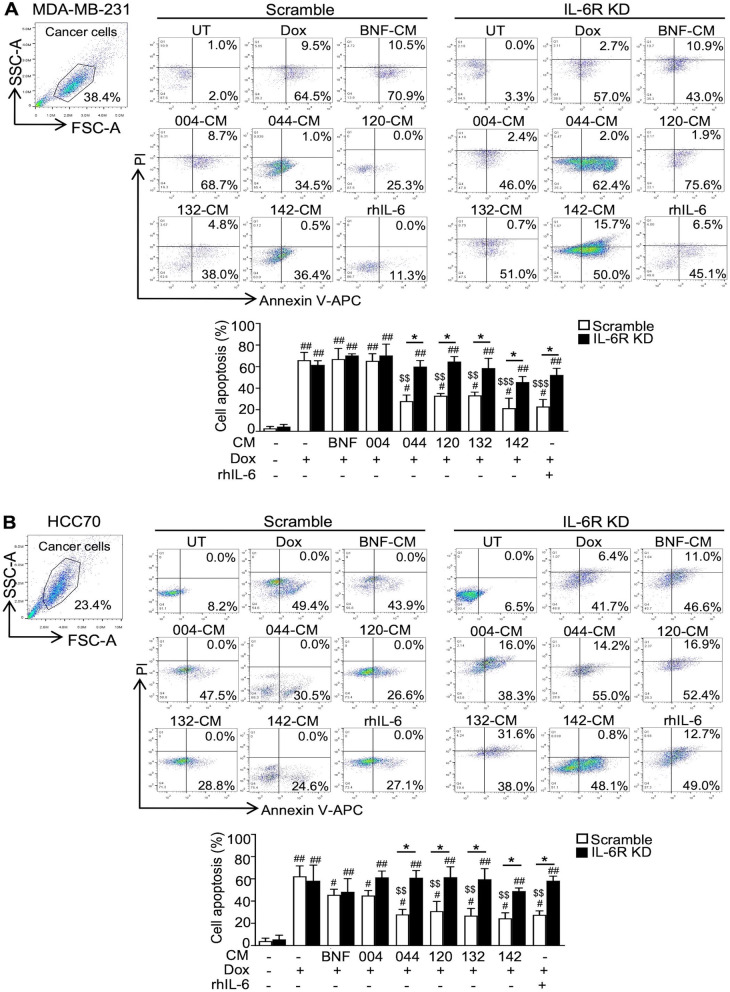
Effect of CAF-derived IL-6 on Dox-induced apoptosis. BCA cell lines after incubation in the presence of CM with or without Dox (5 uM) for 48 h. **A** The apoptosis of MDA-MB-231 and **B** HCC70 after staining with the Annexin V-APC/PI and analyzing by flow cytometry. The representative dot plots of cell apoptosis and the percentage of cell apoptosis in the bar graph (means ± SD of three independent experiments) are shown, ^#^*P* < 0.05, ^##^*P* < 0.01 compared to CM-free and Dox-free condition using one-way ANOVA, ^$$^*P* < 0.01, ^$$$^*P* < 0.001 compared to CM-free with Dox condition using one-way ANOVA, *P < 0.05 compared within the group using Student's *t* test. FSC-A, forward scatter area; SSC-A, side scatter area; UT, untreated cells; Dox, doxorubicin; BNF, breast normal fibroblast; CM, conditioned medium; IL-6R KD, IL-6R knocked down; rhIL-6, recombinant human IL-6

Dox significantly induced apoptosis of scramble MDA-MB-231 cells compared to untreated cells (*P* < 0.01) (Fig. 3A). Low IL-6 PC-B-004-CM and BNF-CM pretreated cells showed the same level of apoptotic cells to no CM-pretreated cells. Interestingly, high IL-6 CMs stimulated Dox-induced apoptosis (28.0 ± 5.7% for PC-B-044 CM, 33.0 ± 2.0% for PC-B-120 CM, 33.3 ± 3.1% for PC-B-132 CM, and 21.5 ± 9.2% for PC-B-142 CM, *P* < 0.05), but this was significantly lower cell apoptosis than that of low IL-6 CM treatment. This implies that high IL-6 CM reduced cell sensitivity to Dox-induced apoptosis. In IL-6R KD cells, the sensitivity to Dox could significantly restore to the same level of Dox-treated scramble cells (*P* < 0.01) and significantly increased compared to the apoptotic cells of high IL-6-CM-treated intact IL-6R cells (*P* < 0.05). Similar results of high IL-6-CM reduced Dox-induced apoptosis of HCC70 cells and the restoration of drug sensitivity in the impaired IL-6R HCC70 cells were observed (Fig. 3B). These findings indicate that CAF-derived IL-6 increases Dox resistance partly through IL-6/IL-6R interaction.

### CAF-derived IL-6 induced PD-L1 expression via STAT3 and AKT signaling pathways

**Fig. 4:**
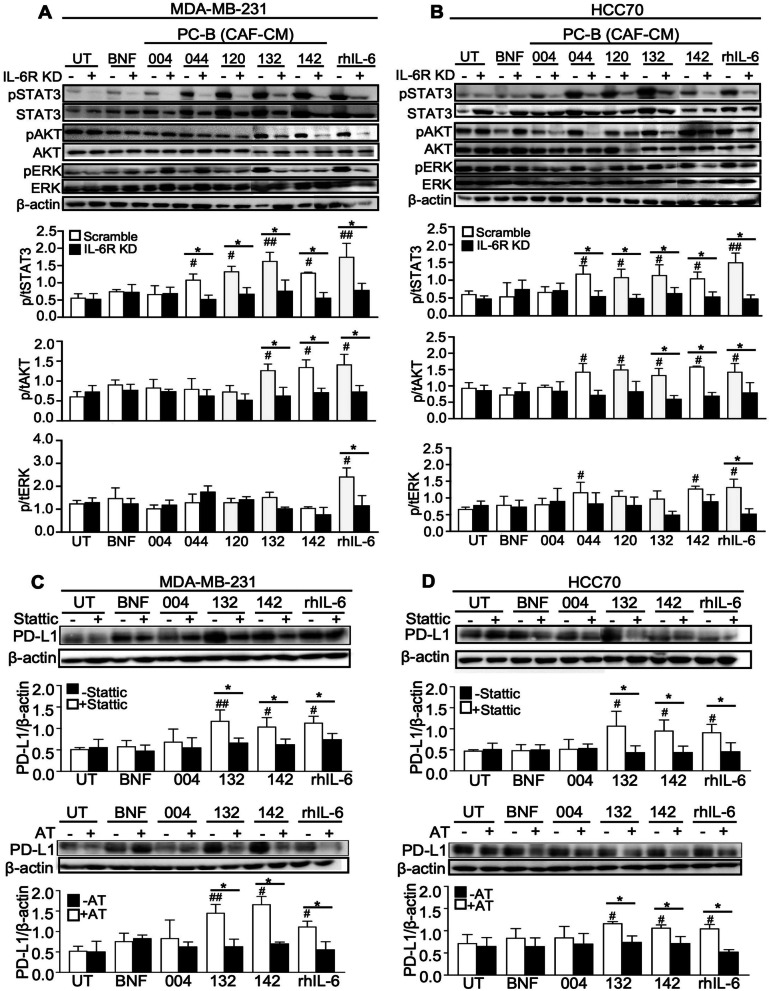
Effect of CAF-derived IL-6 on PD-L1 expression through STAT3 and AKT pathways and the blockage with specific inhibitors. **A** Scramble and si*IL-6R* KD cells after pre-treating with CM for 30 h to observe the phospho- and total protein levels of STAT3, AKT, and ERK using Western blot analysis in MDA-MD231 and **B** HCC70 cell lines. The levels of phosphoproteins were normalized against relative total protein and *β*-actin. **C** The level of PD-L1 of MDA-MD231 and **D** HCC70 following blocking with STAT3 (Stattic) or AKT (AT) inhibitors for 2 h. *β*-actin was used for semiquantitative analysis. Bar graphs represented as mean ± SD of 3 independent experiments. ^#^*P* < 0.05, ^##^*P* < 0.01 compared to BNF using one-way ANOVA, *P < 0.05 compared within group using Student's *t* test. UT, untreated cells; BNF, breast normal fibroblast; CAFs, cancer-associated fibroblasts; CM, conditioned-medium; IL-6R KD, IL-6R knocked down; rhIL-6, recombinant human IL-6

For MDA-MB-231, pSTAT3 was significantly induced by all high IL-6 CM (PC-B-044, PC-B-120, PC-B-132, and PC-B-142), while pAKT was activated by only PC-B-132 and PC-B-142 (*P* < 0.05) (Fig. 4A). In the CM-treated IL-6R KD cells, pSTAT3, pAKT, and pERK had no significant upregulation compared to those of scramble MDA-MB-231 and HCC70 cells. This induction was attenuated when cells had impaired IL-6R expression. The same pattern of results was observed in HCC70 cells (Fig. 4B). In addition to pSTAT3 and pAKT, rhIL-6-treated cells could induce pERK in both cells (Fig. 4A, B). The results suggest that high IL-6-CM activated pSTAT3 and pAKT signaling pathways, but no changes of pERK, probably via IL-6R.

Without Stattic or AT inhibitors, PD-L1 in cells treated with low IL-6 CM (PC-B-004) showed no significant differences compared to that of BNF-CM treatment, whereas high IL-6 CM (PC-B-132 and PC-B-142) significantly induced PD-L1 (*P* < 0.05) in MDA-MB-231 (Fig. 4C) and HCC70 (Fig. 4D). Blocking with Static or AT inhibitors significantly decreased the PD-L1 levels of high IL-6-CM treatment compared to without inhibitors in both cells.

### CAF-derived IL-6 compromised FR*α*-CAR T cell killing ability

**Fig. 5:**
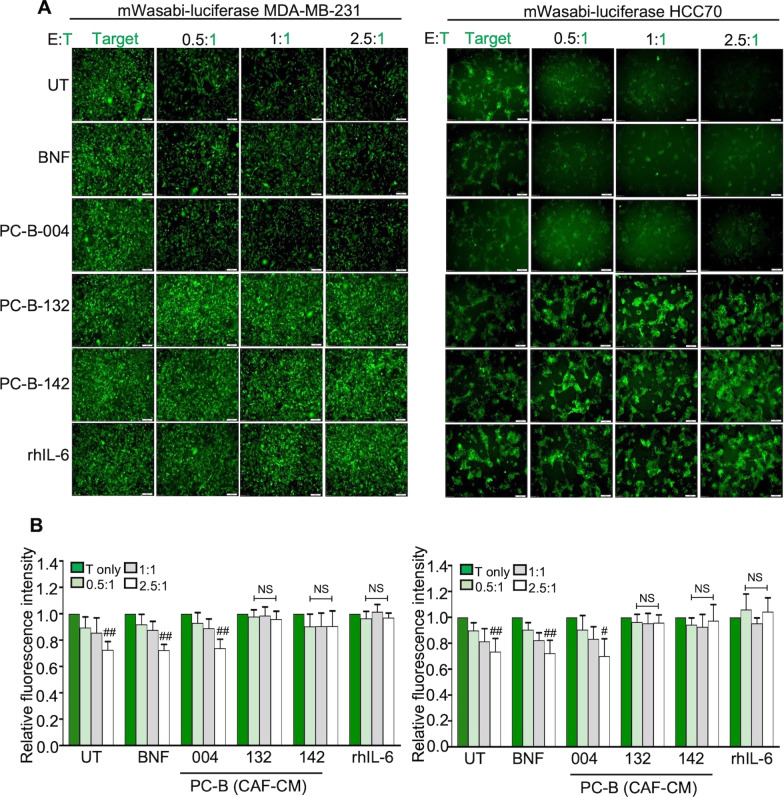
CAF-derived IL-6 compromised FR*α*-CAR-T cell killing ability in 2D cancer cell culture. **A** The representative of fluorescence images shows mWasabi-luciferase expressing target cells (T) after pre-treating with CM and coculturing with effector FR*α*-CAR-T cells (E) at E:T ratios of 0.5:1, 1:1, and 2.5:1 for 24 h (scale bars = 100 μm). The target-only condition (T-only) indicates target cancer cells without coculturing with effector T cells. **B** Bar graphs showing the killing ability of FR*α*-CAR-T cells from 2 donors (mean ± SD of three independent experiments of each donor). ^#^*P* < 0.05, ^##^*P* < 0.01 compared to the T-only condition using one-way ANOVA. UT, untreated cells; BNF, breast normal fibroblast; CAFs, cancer-associated fibroblasts; CM, conditioned-medium; IL-6R KD, IL-6R knocked down; rhIL-6, recombinant human IL-6; NS, no significance

**Fig. 6:**
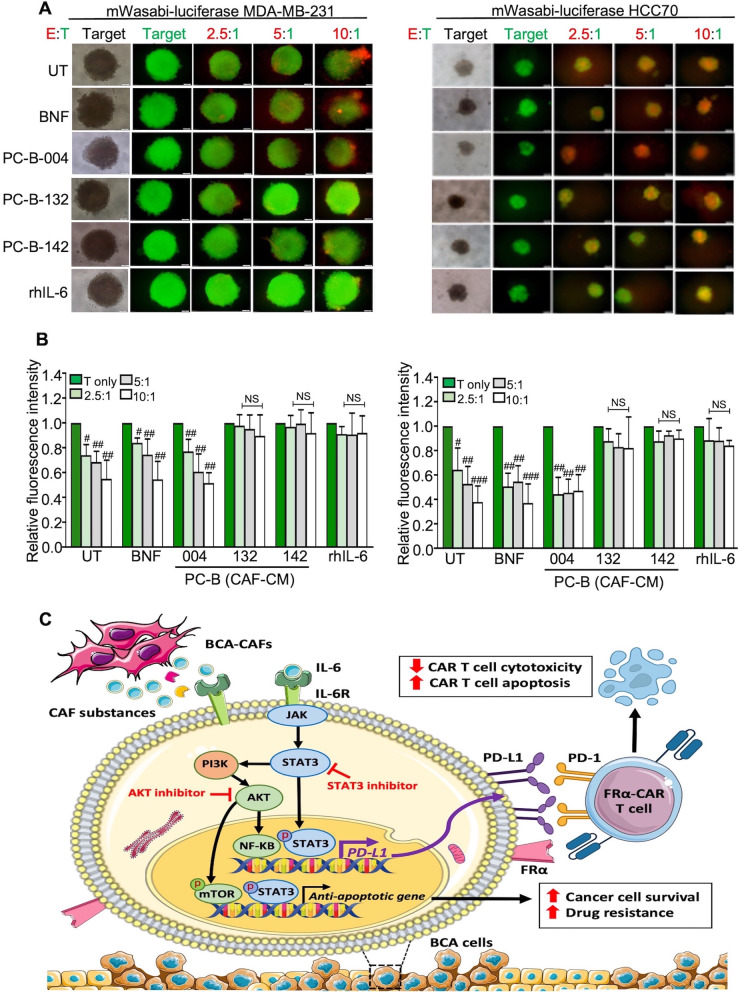
CAF-derived IL-6 attenuated FR*α*-CAR T cell cytotoxicity in 3D cancer cell culture. **A** The representative of spheroids of mWasabi-luciferase expressing target cells (T) after pre-treating with CM and coculturing with effector FR*α*-CAR T cells (E) at E:T ratios of 2.5:1, 5:1, and 10:1 for 4 days (scale bars = 100 μm). The target-only condition (T-only) indicates target cells without effector cells. **B** Bar graphs show the killing ability of FR*α*-CAR-T cells from two donors (means ± SD of three independent experiments of each donor). ^#^*P* < 0.05, ^##^*P* < 0.01, ^###^*P* < 0.001 compared to the T-only condition using one-way ANOVA. **C** The proposed mechanism of CAF-derived IL-6 in promoting resistance to chemo- and immunotherapy. CAFs secrete high IL-6 which can bind to IL-6R on BCA cells. CAF-derived IL-6 induces Dox resistance by promoting cancer cell survival and promoting PD-L1 production through STAT3 and AKT signaling pathways. This PD-L1 induction by CAF-derived IL-6 can be attenuated by STAT3 and AKT inhibitors. The high PD-L1 expressing cancer cells suppress FR*α*-CAR-T cell killing ability. UT, untreated cells; BNF, breast normal fibroblast; CAFs, cancer-associated fibroblasts; CM, conditioned medium; IL-6R KD, IL-6R knocked down; rhIL-6, recombinant human IL-6; NS, no significance. BCA; breast cancer, FR*α*; folate receptor alpha, PD-L1; programmed death ligand 1, PD-1; programmed death 1, CAR T cell; chimeric antigen receptor T cell

The cancer cell killing ability of FR*α*-CAR T cells in 2D (Fig. 5) and 3D cancer cell culture (Fig. 6) was observed. In the 2D killing assay, coculturing of FR*α*-CAR-T cells (E) with FR*α* expressing-target cancer cells (T), MDA-MB-231 and HCC70 cells (Additional file 2: Fig. S2) at E:T of 0.5:1, 1:1, and 2.5:1 reduced fluorescence intensity of viable target cells, with a significant reduction level at only 2.5:1 (*P* < 0.01) (Fig. 5A, B). A similar capability of FR*α*-CAR-T cells was observed in low IL-6 CM (BNF, PC-B-004)-treated cells in both MDA-MB-231 and HCC70 cells (*P* < 0.05) (Fig. 5A, B). In contrast, both MDA-MB-231 and HCC70 cells exposed to high IL-6 CM (PC-B-132 and -142) showed resistance to FR*α*-CAR-T cell killing activity. Similar results were exhibited in the 3D killing assay in the same manner (Fig. 6A, B). Interestingly, in 3D culture system, FR*α*-CAR T cells and cancer cells coculture reduced the viability of cancer cells in a dose-dependent manner with statistical significance.

## Discussion

CAFs produce many substances such as IL-6, IL-8, and TGF-*β* impacting cancer progression and immunosuppression [6–9, 14]. TGF-*β* activated CAFs to secrete IL-6 resulting in the enhancement of CRC metastasis [28]. IL-6 is a major factor of BCA-CAF secretion that can induce resistance to anti-estrogens [29]. In HCC, CAFs were the major source of IL-6 [30]. High IL-6 expression CAFs greatly decreased CD8^+^ T cell infiltration in tumors [30]. CAFs-derived IL-6 induced pancreatic cancer cell migration and invasion [31]. CAF-secreted IL-6 reduced NK cells' function in CRC cells by promoting monocyte differentiation into M2 macrophages and recruiting to the tumor [32, 33]. No investigation, however, has assessed the role of CAF-derived IL-6 in T cell function regulation via induction of PD-L1 in BCA. In this current study, CAF-derived IL-6 exhibited a significantly higher level among other cytokines. IL-6 releases into TME and may be involved in cancer progression, immunosuppression, and the responses of cancer cells to chemotherapy and immunotherapy.

CAFs were positive for vimentin (VIM) and alpha-smooth muscle actin (ASMA), but negative for cytokeratin (CK) expression ensuring that CAFs culture is not contaminated by cancer cells [13]. The immunosuppressive role of FAP^+^ CAFs has been extremely studied in various types of cancers such as head and neck, pancreatic, breast, lung, and liver cancers [13]. In head and neck cancer, FAP^+^ CAFs inhibited CD8^+^ T cell proliferation and promoted regulatory T cell (Treg) recruitment by secreting TGF-*β* and IL-6 [34]. In the BCA mouse model, the elimination of FAP^+^ CAFs showed an increasing CD8^+^ T cell population [35]. IL-6 from CD10^+^ GPR77^+^ CAFs promoted tumor formation in lung cancer [27, 36]. CD10^+ ^GPR77 ^+^ CAFs enriched a survival niche for cancer stem cells (CSCs), enhanced tumor formation, and induced cancer chemoresistance by secreting IL-6 leading to driving the activation of NF-κB via p65 phosphorylation and acetylation, which maintained by complement signaling via GPR77 [27]. Targeting the CD10^+^GPR77^+^CAFs subset retards tumor formation and reverses chemoresistance by destroying the CSC niches [27, 36]. CD10^+^ GPR77^+^ FAP^+^ CAFs were associated with chemoresistance and overall survival of advanced gastric cancer patients who underwent neoadjuvant chemotherapy (NCT) [37]. FAP increasing drug resistance are various, such as promoting immunosuppression and producing chemokine [37]. CD10 and GPR77 have been proven to promote cancer formation and chemoresistance by providing a survival niche for CSCs and promoting an epithelial-mesenchymal transition [37]. In addition, CAFs affect the chemotherapy through an increased interstitial fluid pressure forming a barrier to the transcapillary transport of drugs from blood vessels to cancer cells [38]. All mentioned evidence supports the present results that high IL-6-secreting CAFs were positive for CD10^+^ GPR77^+^ FAP^+^ which may correlate with drug resistance and may regulate anti-tumor immune responses.

In addition to the IL-6 derived from CAFs, IL-6 can be secreted from BCA cells which then promotes cancer cell metastasis in an autocrine effect [39, 40]. Cancer cell-derived IL-6 acted to promote tumorigenesis in BCA, HCC, and lung cancer [41, 42]. Secreted IL-6 binding to membrane IL-6R induces STAT3 expression which is aberrantly active in BCA to promote cancer proliferation and anti-apoptosis [43, 44]. Moreover, STAT3 cooperates with NF-κB in IL-6 induction and the IL-6/STAT3 pathways synergize in the induction of c-MYC leading to promoting cancer cell survival [45].

In contrast to the tumor-promoting effect of IL-6 through the classical IL-6 signaling via membrane IL-6R and glycoprotein 130 (gp130) subunit, in some cancers, IL-6 trans-signaling which is a complex of IL-6 and the soluble IL-6R and membrane gp130 [46], but not IL-6 classic signaling, is mandatory for the development of liver carcinogenesis [46]. Therefore, specific inhibition of IL-6 trans-signaling, rather than total inhibition of IL-6 signaling, is sufficient to blunt tumor initiation and impair tumor progression without compromising IL-6 classic signaling-driven protective immune responses.

CAFs contribute to chemoresistance and immunotherapeutic resistance by upregulating immune checkpoint molecules [22, 23]. The CAF-derived IL-6 promoted PD-L1 expression via STAT3 and AKT pathways and specific inhibitors against pSTAT3 and pAKT attenuated PD-L1 expression induction by IL-6-derived CAF-CM. HCC-CAFs-induced PD-L1^+^ neutrophils through the IL6-STAT3 pathway involved in immunosuppression were previously reported [22]. Moreover, the activation of the STAT3-PI3K/AKT pathway leads to high expression of PD-L1 in various types of cancers [47]. PD-L1 expression is transcriptionally regulated by STAT3 and MYC [48]. The pSTAT3 dimers directly bind on the PD-L1 gene promoter inducing its expression [49] which has been detected in non-small cell lung cancer [50] and head and neck squamous cell carcinoma [51]. The immunosuppressive PD-1/PD-L1 pathway participates in cancer immune escape via the abnormal activation of the tumor-intrinsic STAT3-PI3K/AKT pathway leading to high expression of PD-L1 resulting in attenuation of anti-tumor T cell responses. Interestingly, IL-6 pathway inhibitors are currently tested in combination with chemotherapy in patients with BCA (NCT03135171), pancreatic cancer (NCT04258150, NCT02767557), or liver cancer (NCT04338685), who displayed potent anti-cancer activity with a low incidence of IL-6 drug toxicity [6]. The PD-L1 expression decreased by hesperidin through inhibition of AKT and NF-κB signaling pathways in the MDA-MB-231 cell line [19]. These reports confirm present findings that IL-6 can inhibit antitumor immunity by promoting PD-L1 expression via STAT3 and AKT pathways.

It is suggested that targeting the IL-6/IL-6R/STAT3 axis is expected to become an important strategy for BCA treatment [52]. IL-6 acts directly on cancer cells to induce the expression of STAT3-encoding proteins driving cancer proliferation, i.e., cyclin D1 and survival, i.e., BCL2-like protein 1 (BCL-xL). Early-phase trials exploring the safety and effectiveness of tocilizumab (anti-IL-6RmmAb) in BCA patients are currently ongoing (NCT03135171) [52]. Some high IL-6-containing-CMs, however, did not alter PD-L1 expression. These may be explained by the possibility that CAF secreted various substances which may affect IL-6 secretion by CAFs such as CXCL10 [6, 31]. Current evidence points to chemotherapy-induced DNA damage with nanoparticle albumin bound-paclitaxel (Nab-PTX) influencing the repertoire of CAF-secreted factors after treatment [31]. Nab-PTX treatment increases CXCL-10 expression in pancreatic cancer cells leading to reduced secretion of CAF-derived IL-6 subsequently impairing cancer migration and invasion [31].

DNA damage during chemotherapy is sensed by cyclic GMP-AMP synthase, a stimulator of interferon genes (STING). Dox-mediated DNA damage induced STING-mediated NF-κB activation in TNBC cell lines resulting in the induction of IL-6 secretion which could activate pSTAT3 leading to cancer cell survival enhancement and an immune-suppressive mechanism by PD-L1 expression induction [53]. The cancer cells containing DNA double-strand breaks increased IL-6 secretion by DNA damage response proteins such as ataxia-telangiectasia-mutated during oncogene-induced senescence [54]. Moreover, IL-6 could induce ATM kinase leading to DNA damage repair [55].

Moreover, CAFs reprogramming by vitamin A and vitamin D were shown to inhibit tumor-supportive secretomes [6, 56]. Vitamin A and vitamin D reprogramming strategies are both associated with TGF-*β* signaling pathway inhibition. Indeed, vitamin D decreases fibroblast activation [57], whereas vitamin A inhibits the fibroblasts' capacity to release active TGF-*β*, thus impeding CAF-secreted factors that are associated with the chemo- and immunotherapeutic resistance [58]. Additional research with a better understanding of the mechanisms of CAF secreted-substances that affect IL-6 secretion is needed to confirm.

Importantly, the previous studies of IL-6 in animal models support our findings that IL-6 mediated drug resistance and induced PD-L1 expression. Activation of IL-6 inflammatory loop-induced trastuzumab resistance in breast cancer mouse xenografts [59]. Using a Bcl-2 antagonist (sabutoclax) could inhibit the IL-6/STAT3 signaling to resensitize Dox-resistant breast cancer in vitro and in vivo models [60]. Blocking of STAT3 can prolong the survival time of tumor-bearing mice by suppressing tumor growth and increasing Dox sensitivity in osteosarcoma [61]. Moreover, the combination of IL-6 blockades and anti-PD-L1 antibodies reduced cancer progression in mouse pancreatic cancer [62].

CAR-T cell immunotherapy has emerged as a cancer therapeutic strategy [63]. FR*α* expression is associated with poor prognosis in BCA patients [64]. Several FR*α*-targeting immunotherapies, such as monoclonal antibody [65] and adoptive T cell therapy [66], have been developed. In the present study, FR*α* expression was found in TNBC cell lines which is consistent with a prior study that reported the high prevalence of FR*α* in TNBC [67]. It was proven that FR*α*-CAR-T cells specifically lysed FR*α*-expressing cells in vitro [25]*.* These current findings indicate that CAF-derived IL-6 increases PD-L1-attenuated CAR-T cell cytotoxicity (Fig. 6C). Targeting CAF-derived cytokines and chemokines in combination with immunotherapies can improve anticancer efficiency such as when targeting the CXCL12-CXCR4 axis reverses FAP + CAF-mediated immunosuppression and synergizes with anti-PD-L1 immunotherapy in pancreatic cancer [68]. In conclusion, therapeutics aim at interfering with the IL6/IL-6R and PD-1/PD-L1 pathways may be combined to improve the response to chemotherapy and cell therapy in TNBC patients.

## Supplementary Information

**Additional file 1.** **Supplement Fig. 1.** CAF-derived substances in different primary culture fibroblasts.

**Additional file 2. Supplement Fig. 2.** Surface FR∝ expression on cell lines and transduction efficiency of FR∝ CAR-induced T cells from human primary T lymphocytes.

## Data Availability

The datasets used and/or analyzed during the current study are available from the corresponding author on reasonable request.
