# Supplementary material for: Enhancement of PD-L1-attenuated CAR-T cell function through breast cancer-associated fibroblasts-derived IL-6 signaling via STAT3/AKT pathways
Source: Breast Cancer Res. 2023 Jul 21;25:86. doi: 10.1186/s13058-023-01684-7 (PMC10362675; doi:10.1186/s13058-023-01684-7)
Supplement: Supplementary file 1 — Additional file 1. Supplement Fig. 1. CAF-derived substances in different primary culture fibroblasts. [file 13058_2023_1684_MOESM1_ESM.docx]

**Breast cancer-associated fibroblast-derived IL-6 increases PD-L1-mediated CAR-T cell function via STAT3/AKT pathways**

Nisa Chuangchot^1,2^, Pranisa Jamjuntra^1^, Supaporn Yangngam^1^, Piriya Luangwattananun^2,3^, Suyanee Thongchot^1,2^, Mutita Junking^2,3^, Peti Thuwajit^1^, Pa-Thai Yenchitsomanus^2,3^ and Chanitra Thuwajit^1*^

1Department of Immunology, Faculty of Medicine Siriraj Hospital, Mahidol University, Bangkok 10700 Thailand
2Siriraj Center of Research Excellence for Cancer Immunotherapy, Research Department, Faculty of Medicine Siriraj Hospital, Mahidol University, Bangkok 10700 Thailand

3Division of Molecular Medicine, Research Department, Faculty of Medicine Siriraj Hospital, Mahidol University, Bangkok 10700, Thailand

**Correspondence:** Chanitra Thuwajit

Department of Immunology, Faculty of Medicine Siriraj Hospital, Mahidol University 10700 Thailand

Email: cthuwajit@yahoo.com

Tel: +66-2-419-6635

Fax: +66-2-418-1636

##### **Supplementary data**

##### **Supplementary Methods**

##### **Protein array analysis of cancer-associated fibroblast secreted substances**

Fibroblast substances from non-tumor fibroblasts (NFs) from normal ovarian tissues and cancer-associated fibroblasts (CAFs) from breast cancer tissues were quantitated using a ProHuman Cytokine Standard 27-Plex assay, Group I (#M50-0KCAF0Y; Bio-Rad, CA) including IL-1β, IL-1Ra , IL-2, IL-4, IL-5, IL-6, IL-7, IL-8, IL-9, IL-10, IL-12p70, IL-13, IL-15, IL-17A, eotaxin, basic fibroblast growth factor (bFGF), granulocyte-colony stimulating factor (G-CSF), granulocyte-monocyte-colony stimulating factor (GM-CSF), interferon-γ (IFN-γ), interferon-γ-induced protein 10 (IP-10), monocyte chemoattractant protein-1 (MCP-1), monocyte inhibiting protein-1α (MIP-1α), monocyte inhibiting protein-1β (MIP-1β), platelet-derived growth factor-BB (PDGF-BB), RANTES, tumor-necrosis factor-α (TNF-α) and vascular endothelial growth factor (VEGF). Briefly, the 24-h fibroblast-conditioned-media (CM) was collected from the cultured 90% confluency CAFs and NFs, centrifuged at 1,000g at 4°C for 10 min, and then determined the concentration of each cytokine/chemokine by the Bio-Plex-200 system (Bio-Rad). The data were analyzed using Bio-Plex-Manager™ Software Version 4.0 (Bio-Rad) and shown as cytokine concentration (pg/ml) represented by mean ± SD. The results were considered statistically significant when *P* < 0.05 by Mann-Whitney test in the GraphPad Prism software version 5 (GraphPad Software Inc., CA).

**Immunocytochemistry staining for fibroblast markers and FRα**

CAFs were fixed with 4% paraformaldehyde, permeabilized in 1% Triton X-100 and blocked with 1% bovine serum albumin (BSA) (Sigma). Cells were incubated with anti-pan cytokeratin (panCK) (1:200, sc-8018; Santa Cruz), anti-vimentin (VIM) (1:500, sc-6260; Santa Cruz), anti-alpha smooth muscle action (ASMA) (1:200, A5228; Sigma), anti-FAP (1:100, ab53066; Abcam) at RT for 3 h; mouse anti-CD10-FITC (1:5, 21270103; Invitrogen) or mouse anti-GPR77 (1:30, 342402; Biolegend) at 4^o^C overnight. The secondary antibodies including anti-mouse IgG-Cy3 (1:2,000, 115-166-071; Jackson ImmunoResearch) or anti-rabbit IgG-FITC (1:2,000, ab6717; Abcam) were applied at RT for 1 h. The nuclei were stained with Hoechst dye (1:1,000; Invitrogen), fixed with 4% paraformaldehyde. The fluorescence signals were captured with the confocal microscope (LSM800, Carl Zeiss Microscopy).

For FRα, TNBC cell lines were stained with anti-human FRα antibody (1:100, LS-B5727; LifeSpan BioSciences, WA). After washing, anti-rabbit IgG-AlexaFlour® 488 (1:1000, A21206; Invitrogen) and Hoechst 33342 (1:5000, H3570; Invitrogen) were added. The stained cells were observed under confocal microscope (LSM800, Carl Zeiss Microscopy). Data were shown as mean ± SD of three independent experiments which was considered statistically significant when *P* < 0.05 analyzed by Student’s *t*-test. All statistical calculations were performed with GraphPad Prism software version 5 (GraphPad Software Inc.).

##### **Supplementary Results**

**CAFs secreted high IL-6 level**

Nine CAF‐CM and 6 NF‐CM were assayed using a cytokine array for 27 cytokines/chemokines including IL‐1β, IL‐1rα, IL‐2, IL‐4, IL‐5, IL‐6, IL‐7, IL‐8, IL‐9, IL‐10, IL‐12p70, IL‐13, IL‐15, IL‐17A, eotaxin (CCL11), FGF, G‐CSF, GM‐CSF, IFN‐γ, IP‐10, MCP‐1, MIP‐1α, MIP‐1β, PDGF‐BB, RANTES, TNF‐α and VEGF. In total, only 10 cytokines, namely IL‐5, IL‐6, IL‐8, CCL11, FGF, GM‐CSF, IFN‐γ, IP‐10, MCP‐1, and VEGF were in the detectable range. Among these 10 cytokines, only IL-6 of the pooled CAF‐CM (397±466 pg/ml) exhibited a significantly increased level compared to pooled NF‐CM (112±78 pg/ml) (*P* < 0.05) (**Supplementary Fig. 1**).


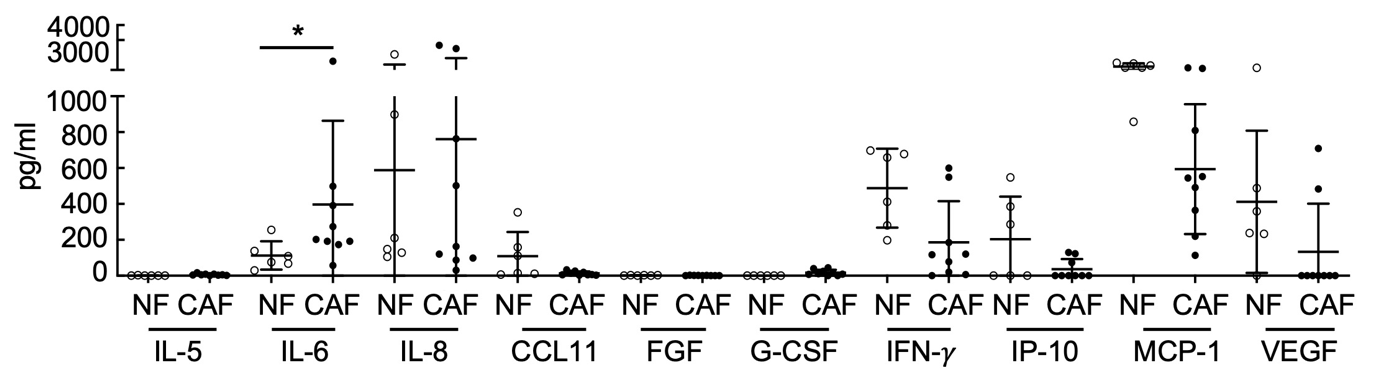


**Supplementary Fig. 1** **CAF-derived substances in different primary culture fibroblasts.** Scatter plots of the secreted substances in 9 pooled CAF-CM and 6 pooled NF-CM. *indicates statistical significance as *P* < 0.05.

**Expression of** FRα **in breast cancer cells and characterization of fourth generation FRα-CAR T cells**

##### **
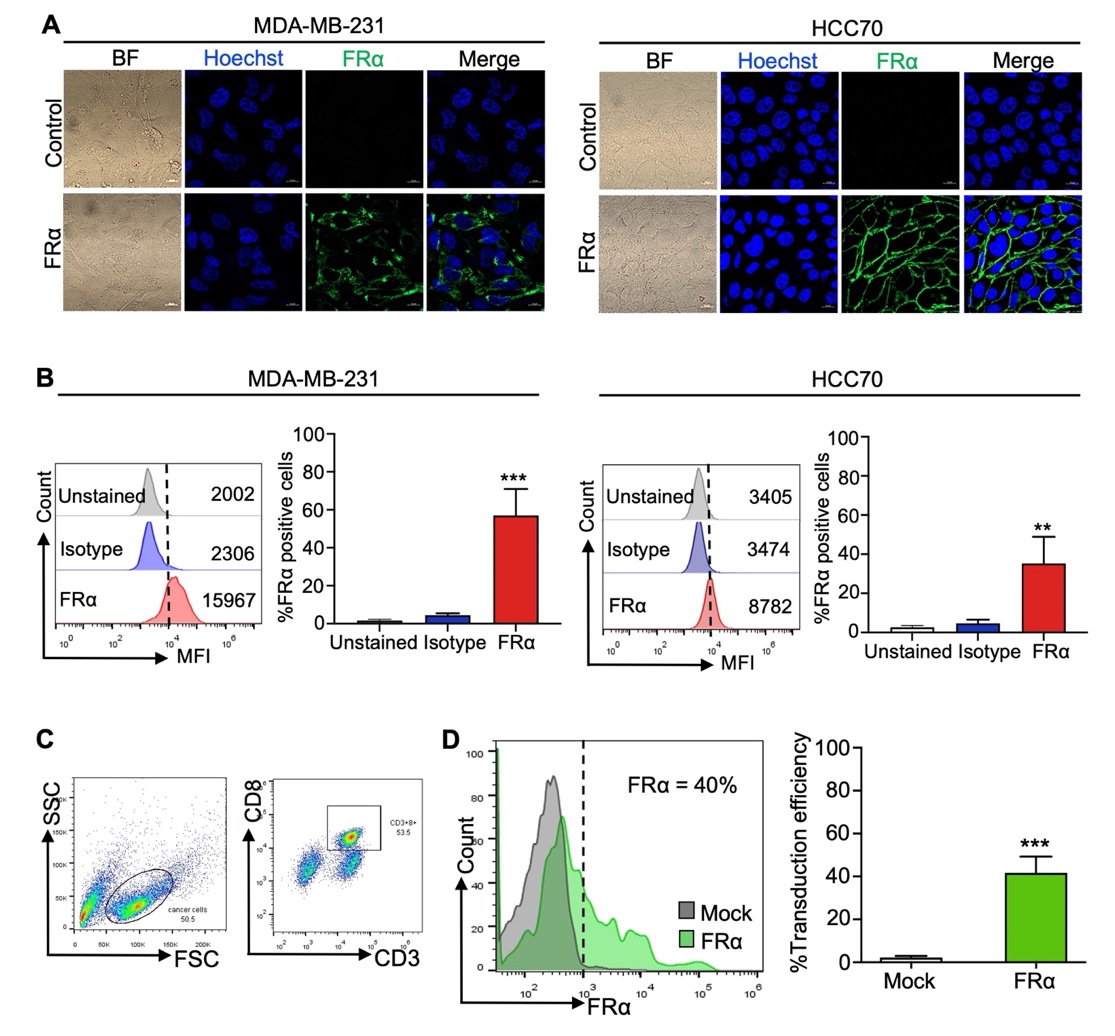
**Confocal images showed the green-fluorescence of FRα expression in MDA-MB-231 and HCC70 cell lines (**Supplementary Fig. 2A**). The surface FRα expression by flow cytometry of MDA-MB-231 cells were 60.0 ± 13.9% (MFI= 15967), while HCC70 cells were 39.0 ± 13.5% (MFI= 8782) (**Supplementary Fig. 2B**). The flow cytometry gating strategy of CD3^+^CD8^+^ expressing-FRα-CAR T cell population was shown as a representative dot plot (**Supplementary Fig. 2C**). The transduction efficacy (**Supplementary Fig. 2D**) which was shown as the representative histogram of CD3^+^CD8^+^ FRα-CAR T cells (green peak of FRα-CAR T cells=40%, grey peak of mock control=0%) and the bar graph cells of 3 independent experiments (FRα-CAR T cells=42.0 ± 7.6% vs. mock control=2.2 ± 0.8%).

**Supplementary Fig. 2 Surface FRα expression on cell lines and transduction efficiency of FRα CAR-transduced T cells from human primary T lymphocytes. (A)** Confocal images showing FRα expression in MDA-MB-231 and HCC70 are shown in green color. Cell nuclei were stained in blue with Hoechst 33342. Scale bars represent 10 μm. BF: bright field. **(B)** Representative histograms from flow cytometry showing FRα expression level in MDA-MB-231 and HCC70. Dashed-lines indicate staining with isotype control antibody, and red-filled histograms indicate staining with anti-FRα antibody. Percentage of FRα-positive cells and mean fluorescence intensity (MFI) of tested to isotype control are indicated with numbers. Percentage of FRα-positive cells summarized from 3 independent experiments. **(C)** Flow cytometry gating strategy of FRα-CAR T cell population in human primary CD3+CD8+ T cells. **(D)** The representative histogram and bar graphs from 3 independent experiments showing the transduction efficiency of CD3+ CD8+ FRα-CAR-transduced T cells and mock transduced T cells. **, *** indicates statistical significance as *P* < 0.01, and < 0.001.
