# Supplementary material for: Enhancement of PD-L1-attenuated CAR-T cell function through breast cancer-associated fibroblasts-derived IL-6 signaling via STAT3/AKT pathways
Source: Breast Cancer Res. 2023 Jul 21;25:86. doi: 10.1186/s13058-023-01684-7 (PMC10362675; doi:10.1186/s13058-023-01684-7)
Supplement: Supplementary file 2 — Additional file 2. Supplement Fig. 2. Surface FR∝ expression on cell lines and transduction efficiency of FR∝ CAR-induced T cells from human primary T lymphocytes. [file 13058_2023_1684_MOESM2_ESM.pdf]

Western blot gel
(Full unedited gel)

Fig 2C: Scramble and IL-6R knock down MDA-MB-231 cell after treating with CAF-CM to detect PD-L1 expression

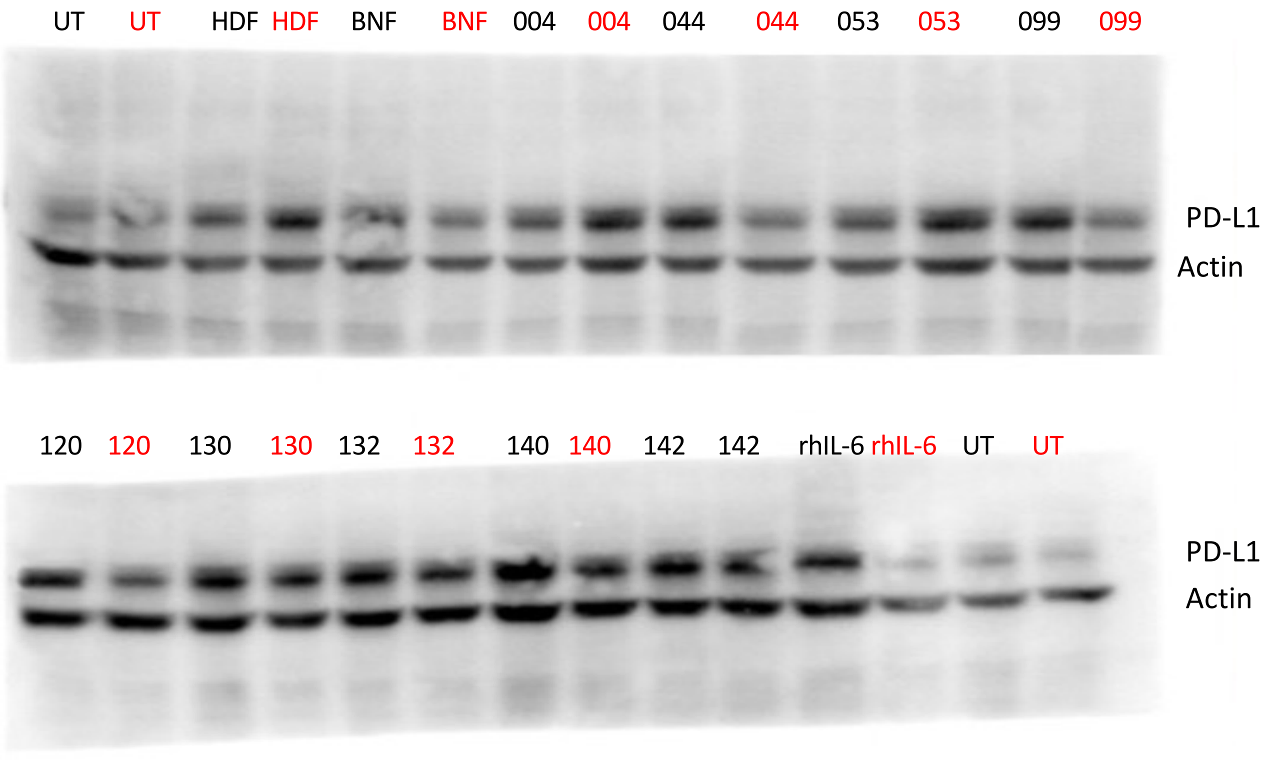

Black font= scramble cell// Red font= IL-6R knock down (KD) cell

Fig 2D: Scramble and IL-6R knock down HCC70 cell after treating with CAF-CM to detect PD-L1 expression

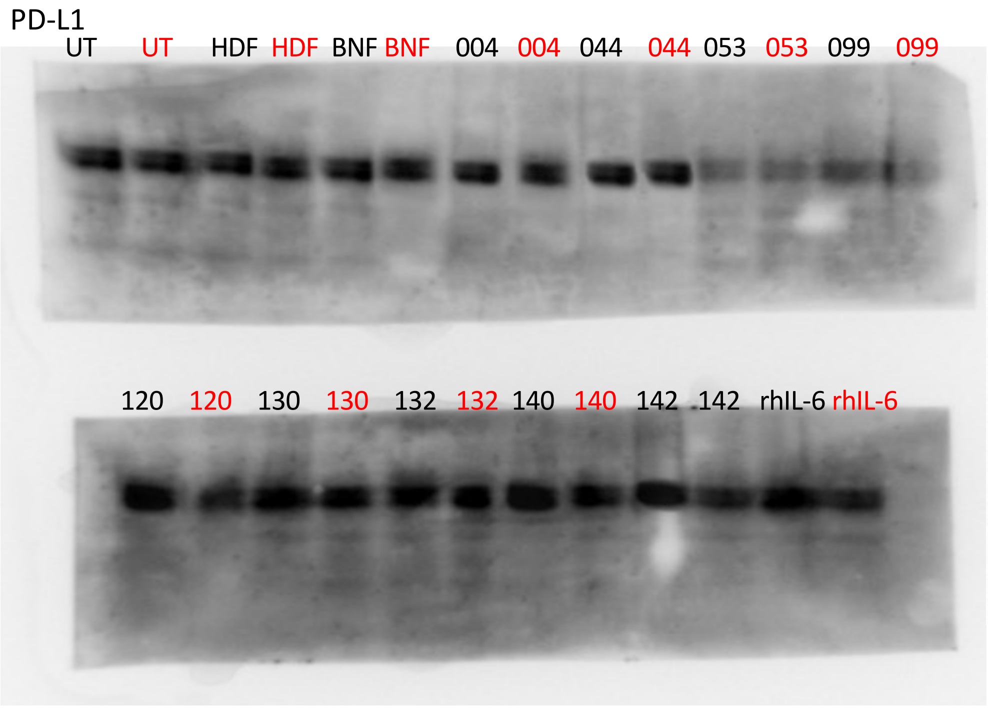

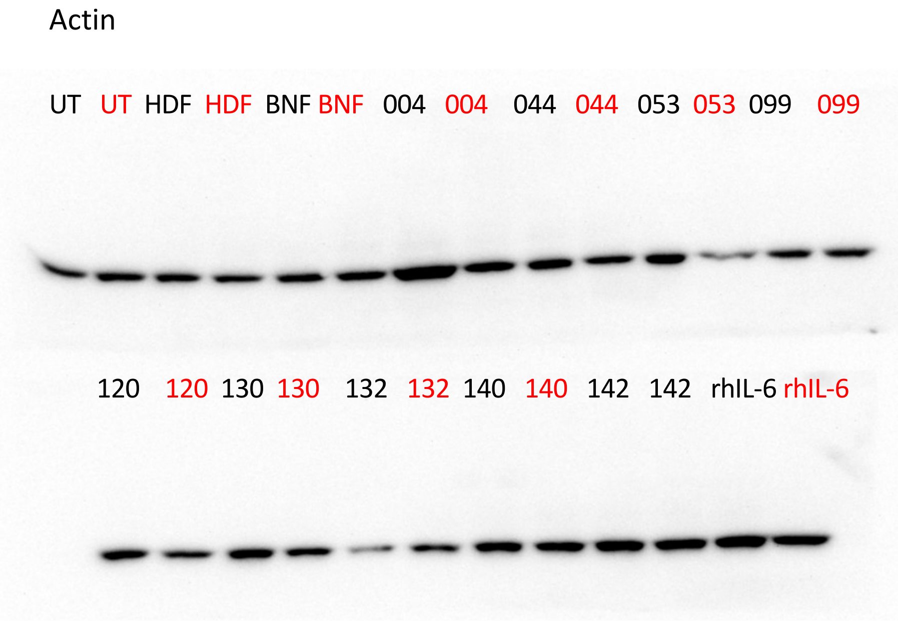

Black font= scramble cell// Red font= IL-6R knock down (KD) cell

Fig 4A: Scramble and IL-6R knock down MDA-MB-231 cell after treating with CAF-CM to detect signaling molecules

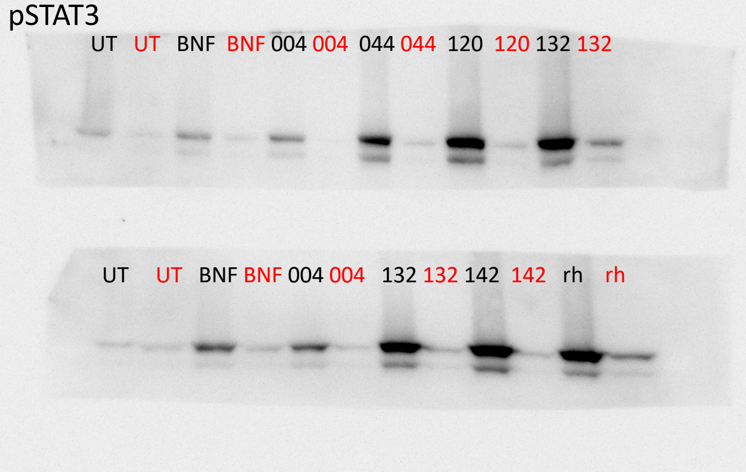

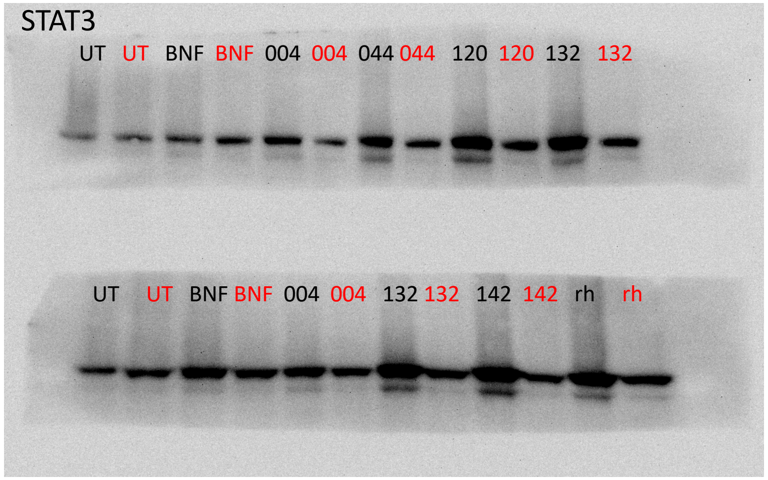

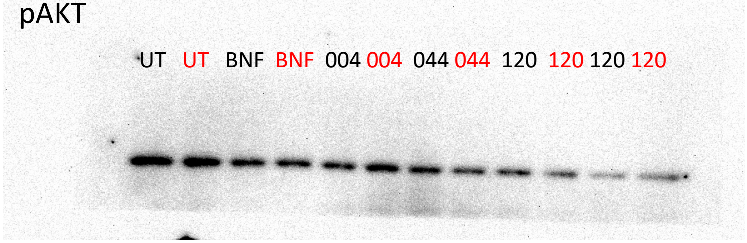

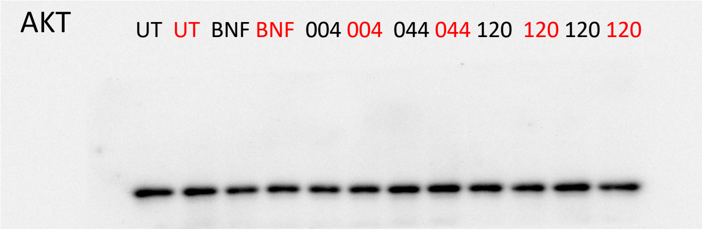

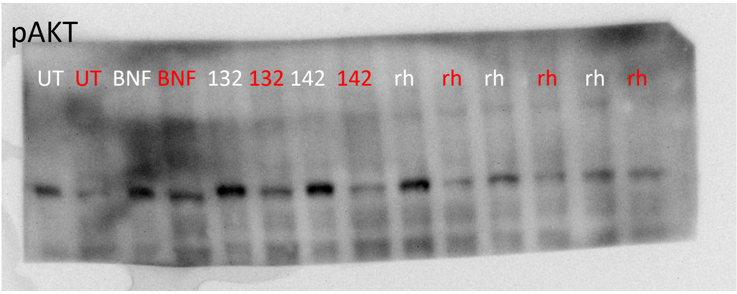

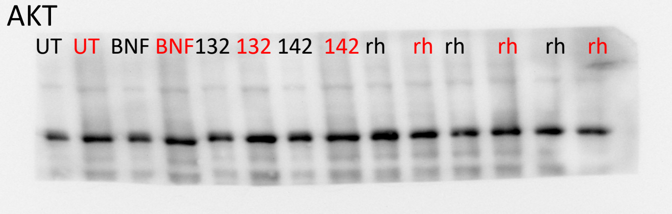

Black font= scramble cell// Red font= IL-6R knock down (KD) cell

Fig 4A: Scramble and IL-6R knock down MDA-MB-231 cell after treating with CAF-CM to detect signaling molecules

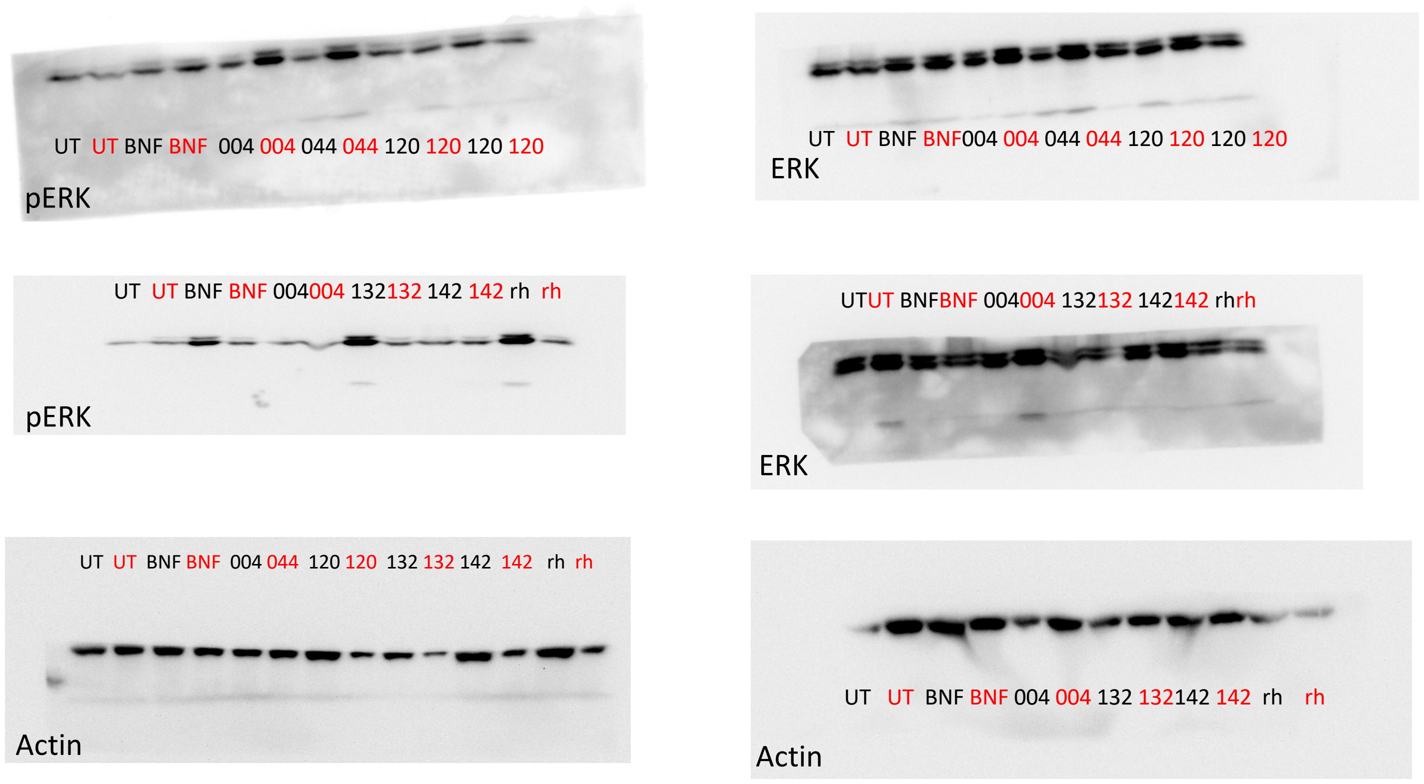

Black font= scramble cell// Red font= IL-6R knock down (KD) cell

Fig 4B: Scramble and IL-6R knock down HCC70 cell after treating with CAF-CM to detect signaling molecules

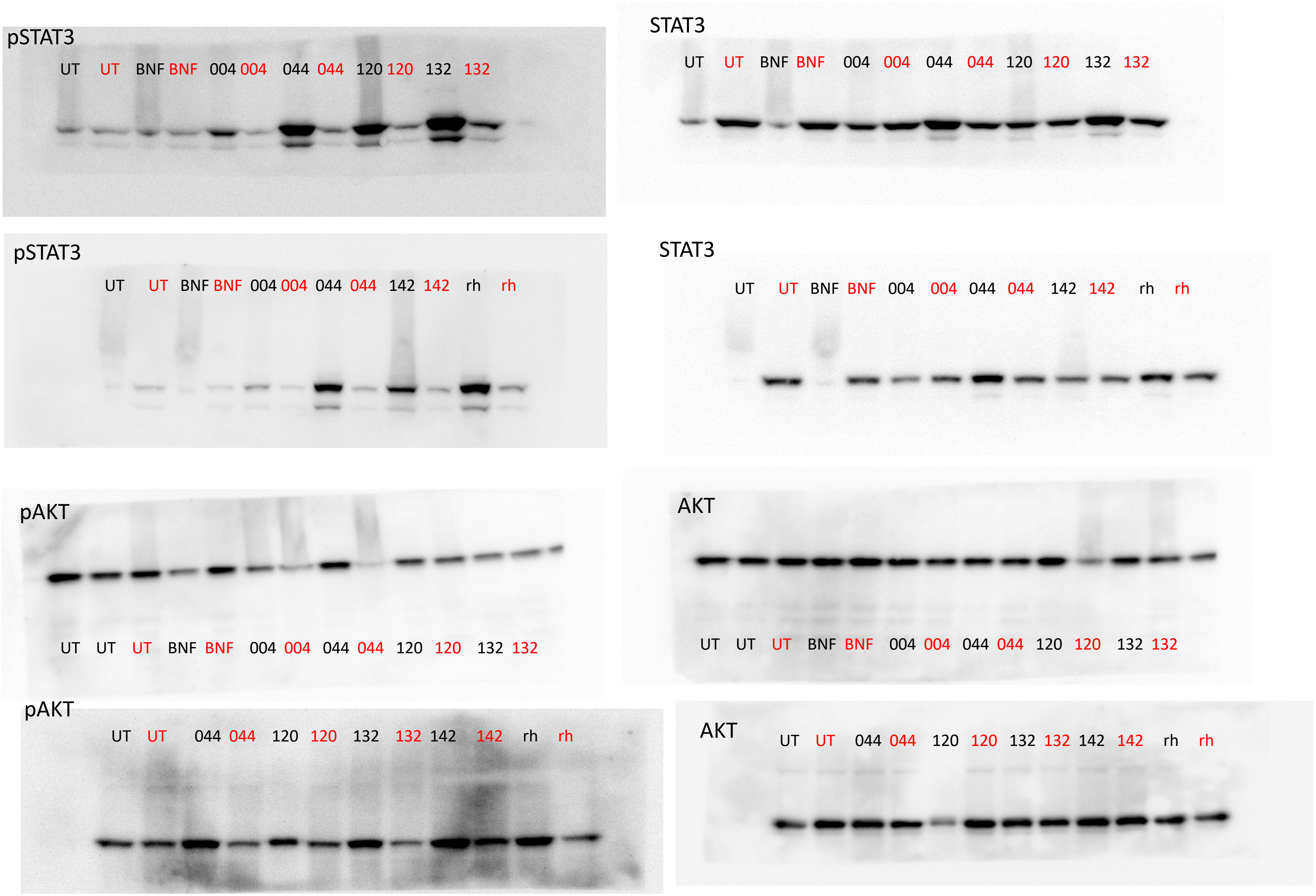

Black font= scramble cell// Red font= IL-6R knock down (KD) cell

Fig 4B: Scramble and IL-6R knock down HCC70 cell after treating with CAF-CM to detect signaling molecules

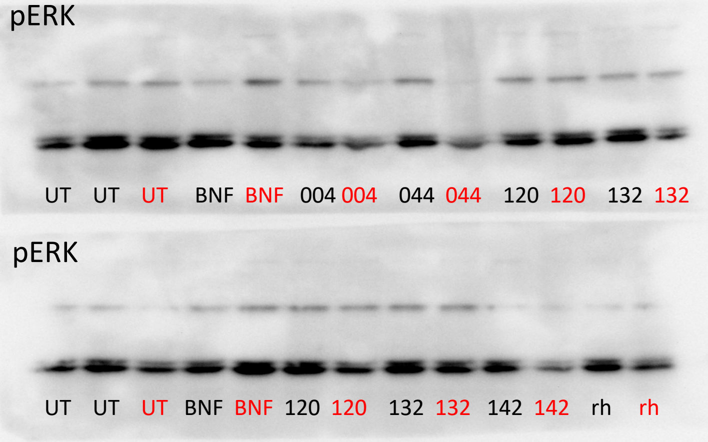

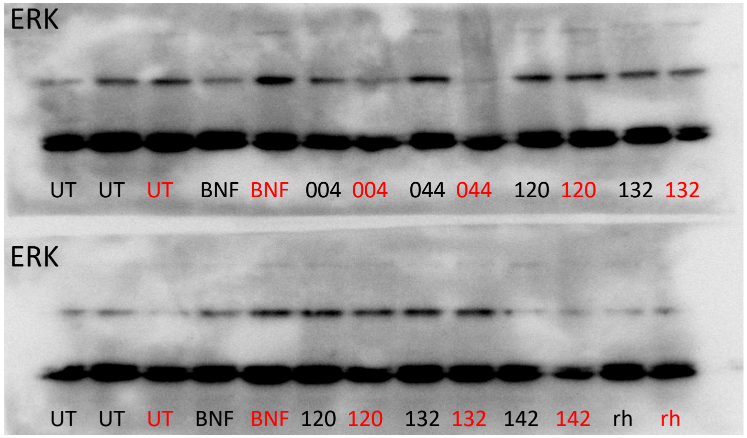

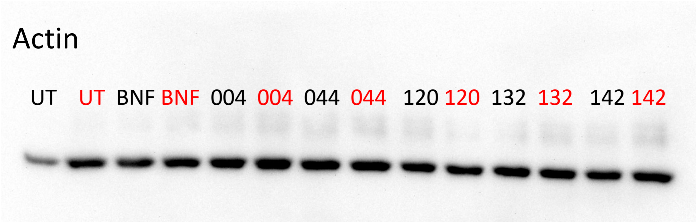

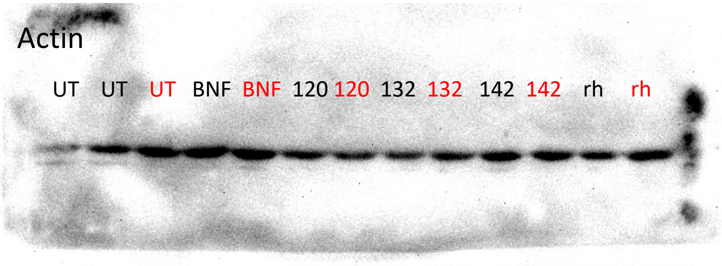

Black font= scramble cell// Red font= IL-6R knock down (KD) cell

Fig 4C: Scramble MDA-MB-231 cell after treating with CAF-CM and specific inhibitors to detect PD-L1 expression

STAT3 inhibitor

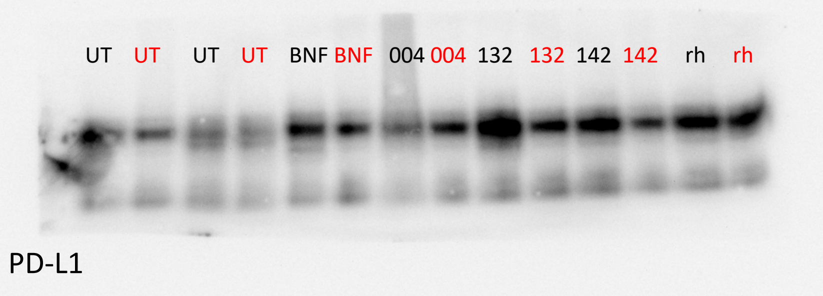

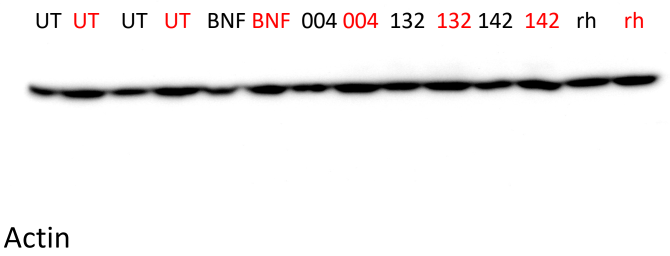

AKT inhibitor

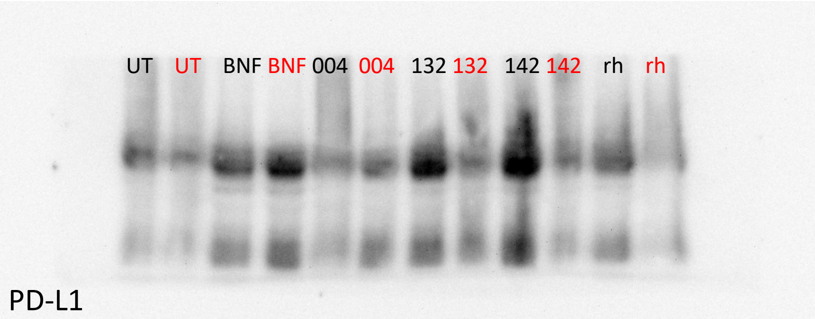

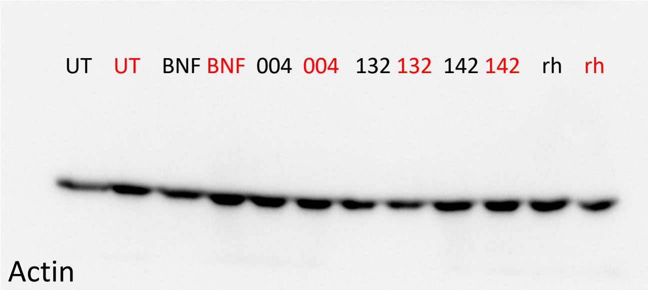

Black font= without inhibitor// Red font= with inhibitor

Fig 4D: Scramble HCC70 cell after treating with CAF-CM and specific inhibitors to detect PD-L1 expression

STAT3 inhibitor

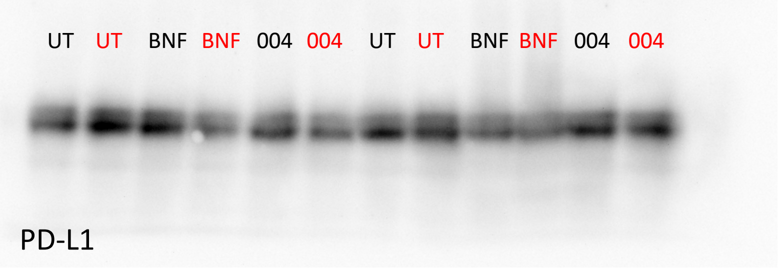

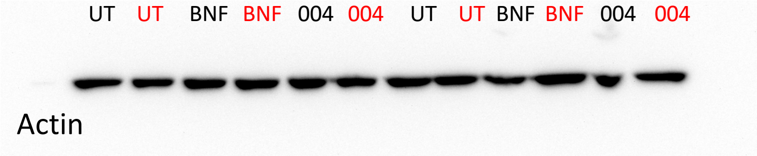

STAT3 inhibitor

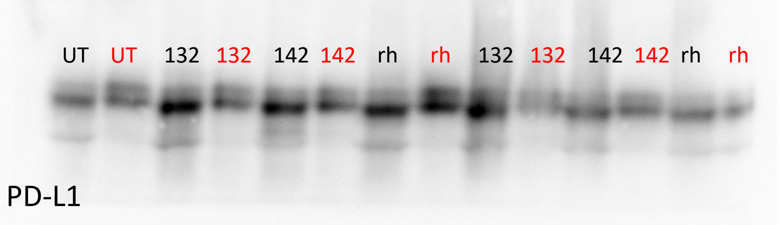

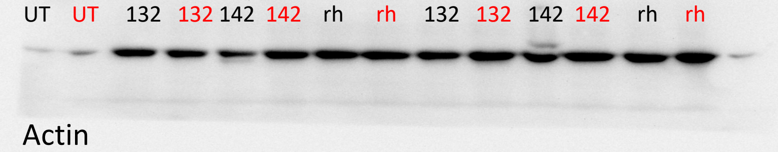

AKT inhibitor

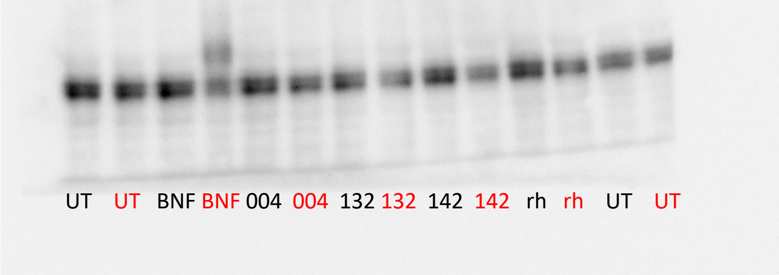

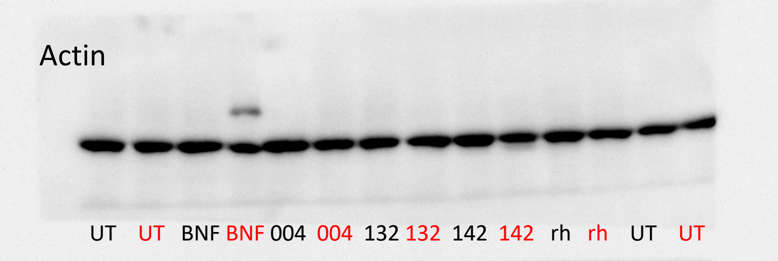

Black font= without inhibitor// Red font= with inhibitor
